# Sleep–neuroinflammation interactions in Alzheimer’s disease: a systematic review of biomarker pathways

**DOI:** 10.3389/fnagi.2026.1854872

**Published:** 2026-09-09

**Authors:** Alberto Cordella, Athina Bruschetti-Thomi, Leonardo Sacco, Claudio Liguori

**Affiliations:** 1 Memory Clinic, Department of Neurology, Neurocenter of Southern Switzerland, Régional Hospital of Lugano, Lugano, Switzerland; 2 Regional Hospital of Lugano, Internal Medicina, Lugano, Switzerland; 3 Faculty of Biomedical Sciences, University of Southern Switzerland, Lugano, Switzerland; 4 Neurology Unit, University Hospital of Rome Tor Vergata, Rome, Italy; 5 Department of Systems Medicine, University of Rome Tor Vergata, Rome, Italy; 6 IRCCS Neuromed, Pozzilli, Italy

**Keywords:** Alzheimer’s disease, astrocytes, biomarkers, circadian rhythms, glymphatic system, microglia, neuroinflammation

## Abstract

Although sleep disturbances are increasingly associated with Alzheimer’s disease (AD), the mechanistic interplay between sleep physiology, neuroinflammation and AD-related pathology is not fully understood. The question of whether disrupted sleep contributes to neurodegeneration or merely reflects early pathological processes is still being debated. In accordance with PRISMA-2020 guidelines, we conducted a systematic review to synthesize the current evidence linking objectively measured sleep alterations, glial activation and AD-related outcomes. A comprehensive search of PubMed, Scopus, EMBASE and Web of Science (January 2000 – June 2026) identified 40 original studies that met the predefined inclusion criteria. These studies included objective sleep assessments (polysomnography, electroencephalography, actigraphy, or experimental sleep manipulation), direct evaluations of neuroinflammatory or glial markers, and assessments of amyloid-*β* (Aβ), tau pathology, neurodegeneration and cognition. Human studies indicate that slow-wave sleep impairment, sleep fragmentation, and REM sleep dysregulation are linked to neuroinflammation and AD-related pathology. Further mechanistic insights come from experimental animal and translational studies, which show that sleep deprivation or disruption may enhance microglial activation, astrocytic reactivity, and the accumulation of AD-related proteins. However, limited prior work has integrated evidence from sleep physiology, central neuroinflammatory measures, and multimodal AD biomarkers in humans. The available evidence supports a biologically plausible, potentially bidirectional relationship between sleep disruption and AD pathology, while highlighting important gaps in translation. Further longitudinal and interventional investigations are required to clarify causality and determine whether targeting sleep physiology could meaningfully influence disease trajectories.

## 1 Introduction

Over the past years, the view of sleep in Alzheimer’s disease (AD) has undergone profound changes. Sleep problems are no longer regarded only as secondary symptoms, but are increasingly recognized as closely associated with disease onset and progression. Increasing data indicate that disrupted slow-wave sleep (SWS), circadian sleep–wake cycle misalignment, and chronic sleep loss may contribute to molecular and cellular processes linked to neurodegeneration (Heneka et al., 2023; Irwin, 2019; Lucey et al., 2019; Mander et al., 2016). These alterations are linked to inflammatory activation and impaired clearance mechanisms, potentially promoting the accumulation of amyloid-*β* (Aβ) and tau and affecting synaptic function and cognitive stability.

In physiological conditions, sleep plays a fundamental role in supporting the clearance of interstitial solutes and metabolic waste. During SWS, glymphatic flow promotes the exchange between cerebrospinal and interstitial fluids, facilitating the removal of proteins such as Aβ and tau (Rasmussen et al., 2018; Xie et al., 2013). Although evidence in humans remains indirect and evolving, disruption of this process has been associated with reduced clearance efficiency and increased oxidative and inflammatory stress. Microglia and astrocytes, the main immune effectors of the brain, may respond to such imbalance through activation and altered homeostatic functions, releasing cytokines and modifying synaptic microenvironments (Bubu et al., 2019; Verkhratsky and Nedergaard, 2018; Yirmiya et al., 2015).

In parallel, chronic sleep loss and circadian sleep–wake cycle disruption appear to disturb immune–metabolic signaling. Experimental studies showed that prolonged wakefulness enhances microglial sensitivity and cytokine release, especially IL-1β, IL-6, and TNF-*α*, which are themselves known to influence sleep regulation (Davis and Krueger, 2012; Opp, 2009). Misalignment of the circadian clock further modifies the expression of genes involved in immune control and energy balance, contributing to a persistent low-grade inflammatory tone (Fonken and Nelson, 2015; Musiek and Holtzman, 2019).

Overall, this evidence supports a potentially bidirectional interaction between sleep and neuroinflammation that may begin before the onset of clinical symptoms of AD. Understanding how these systems influence each other may facilitate the identification of early biomarkers and improve our understanding of the earliest stages of AD pathophysiology. Integrating electrophysiological, molecular, and neuroimaging biomarkers may further clarify how alterations in sleep architecture reflect neuroinflammatory processes and the evolution of AD-related pathology.

Previous reviews have addressed individual aspects of the sleep–AD relationship, including disease mechanisms (Lacerda et al., 2024), neuroinflammatory and molecular pathways (Zhang et al., 2025), and associations between sleep characteristics and AD biomarkers (Chen et al., 2025). Building upon these contributions, the present systematic review integrates evidence from experimental, translational, and human studies to jointly evaluate objectively measured sleep disturbances, CNS-relevant neuroinflammatory and glial biomarkers, and AD-related pathological outcomes. Specifically, this review focuses on central neuroimmune mechanisms, including glial activation, cerebrospinal fluid biomarkers, neuroimaging-based markers, and brain tissue-derived inflammatory measures, while not addressing peripheral inflammatory markers in isolation. This integrated approach enables a critical appraisal of the available evidence, identifies current translational gaps, and highlights priorities for future longitudinal and interventional research.

## 2 Methods

### 2.1 Search strategy, study selection and data extraction

The aim of this systematic review was to synthesize evidence on the relationship between objectively measured sleep architecture pathological changes, neuroinflammation and AD, with particular focus on glial activation and AD-related pathological outcomes.

This work was conducted as a systematic review in accordance with the PRISMA 2020 guidelines.

A comprehensive literature search was performed in PubMed, Scopus, EMBASE, and Web of Science to identify articles published between January 2000 and June 2026 addressing the relationship between sleep disturbances, neuroinflammation and AD.

The search strategy combined the following terms in title/abstract fields: *(“sleep” OR “sleep disturbance” OR “insomnia” OR “circadian rhythm” OR “slow-wave sleep”) AND (“Alzheimer’s disease” OR “dementia” OR “amyloid beta” OR “tau”) AND (“neuroinflammation” OR “microglia” OR “astrocytes” OR “inflammatory markers”)*. The systematic review protocol has been registered in the PROSPERO database (PROSPERO ID 1365834 Available from https://www.crd.york.ac.uk/PROSPERO, edited on April 10, 2026). The last search was conducted on June 10th 2026. All retrieved records were exported into Rayyan, and duplicates were removed using a combination of automated and manual screening prior to title/abstract review.

**Figure 1:**
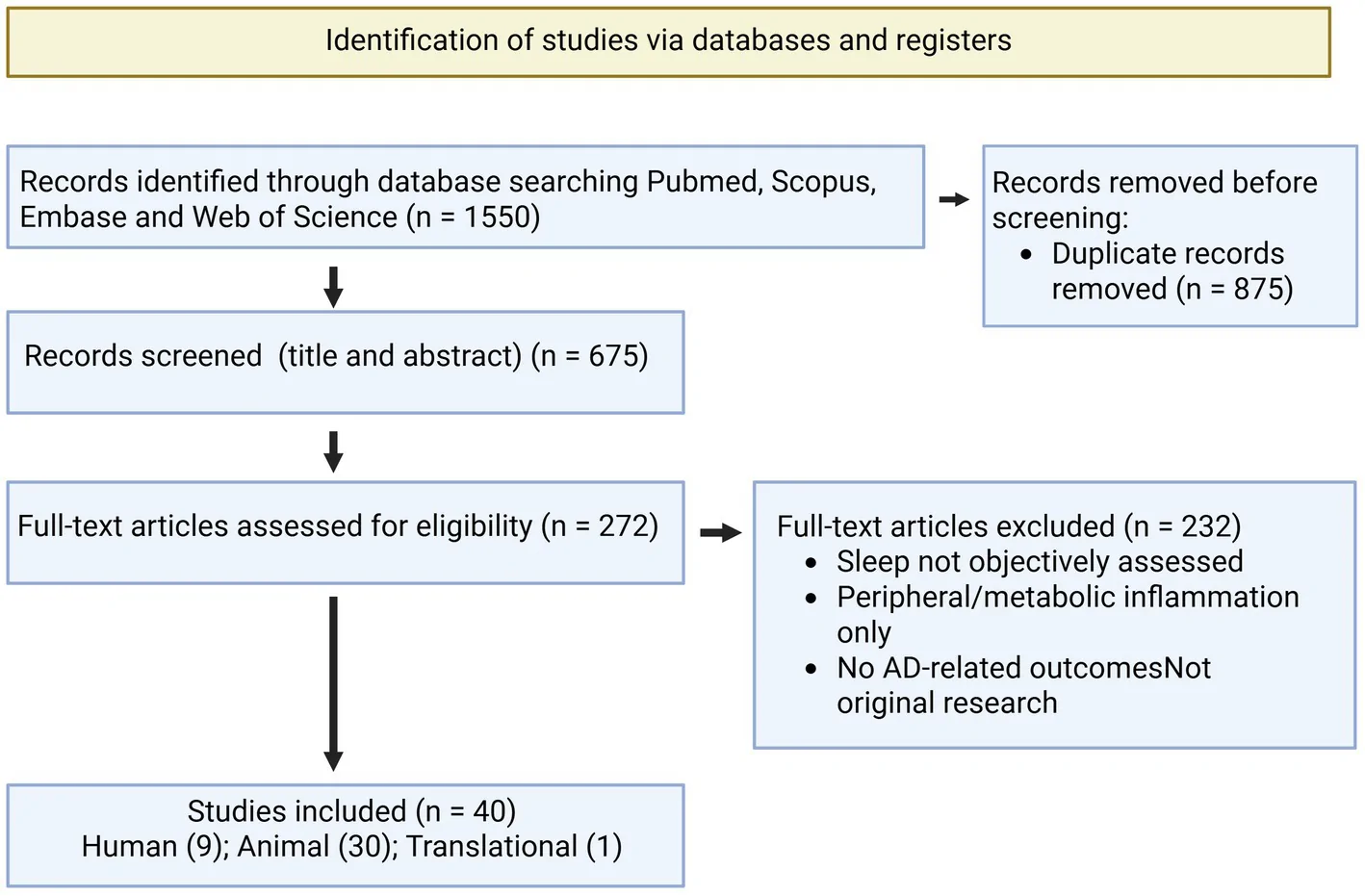
Flow diagram illustrating the identification, screening, eligibility assessment, and inclusion of studies in this review. A total of 1,550 records were identified through database searching. After duplicate removal, 675 records were screened and 272 full-text articles were assessed for eligibility. 232 studies were excluded for not meeting predefined inclusion criteria. Forty original studies (9 human, 30 animal and 1 mixed/translational) were included in the final qualitative synthesis. Created by the authors using BioRender.

The study selection process is summarized in the PRISMA-2020 flow diagram (Figure 1).

Eligibility criteria were defined according to the PICOS framework. The population included human participants across the AD continuum, as well as animal and translational models relevant to AD pathology. The exposure of interest consisted of objectively measured sleep architecture or experimental sleep manipulations, including polysomnography, electroencephalography, actigraphy, sleep deprivation, sleep restriction, sleep fragmentation, and circadian disruption paradigms. Comparator groups included healthy controls, normal sleep conditions, or study-specific comparator groups when available. Outcomes of interest comprised central or CNS-relevant neuroinflammatory biomarkers, glial markers, AD-related biomarkers (including Aβ and tau measures), neurodegenerative outcomes, and cognitive measures relevant to AD. Eligible study designs included original observational, experimental, translational, and preclinical studies.

For the purposes of this review, central/CNS-relevant neuroinflammatory or glial biomarkers were operationally defined as measures directly reflecting neuroimmune activity or glial responses within the central nervous system. These included cerebrospinal fluid (CSF) biomarkers (e.g., GFAP, YKL-40/CHI3L1, sTREM2, and inflammatory mediators), neuroimaging-based markers of glial activation (e.g., TSPO-PET), and brain tissue-derived measures of microglial or astrocytic activation and central inflammatory signaling in experimental models. Studies reporting both central and peripheral inflammatory markers were considered eligible only when CNS-relevant measures were available; peripheral-only markers were extracted separately but were not considered sufficient for inclusion.

Titles and abstracts were independently screened for relevance by two reviewers (AC and ABT). Any disagreements regarding study eligibility were resolved through discussion and consensus, involving a third and fourth reviewer (CL and LS) when necessary. Full texts of potentially eligible studies were subsequently assessed according to predefined inclusion criteria.

Studies were eligible if they fulfilled the following criteria:

1) Original research articles;
2) Objective assessment or experimental manipulation of sleep;
3) Direct evaluation of central neuroinflammatory or glial markers;
4) Assessment of AD-related outcomes;
5) English language.

Studies were excluded if sleep was only mentioned as contextual information, if neuroinflammation was assessed exclusively peripherally, if AD-related outcomes were not evaluated, or if the article was not original research.

Given the heterogeneity of study designs, populations, and outcomes, a quantitative meta-analysis was not performed. Findings were synthesized using a structured approach stratified by evidence source (human, animal, and translational studies).

Risk-of-bias assessment was performed according to study design. Human observational studies were evaluated using the Newcastle–Ottawa Scale (NOS), whereas animal and translational studies were assessed using the SYRCLE Risk of Bias tool. Studies were classified as having low, moderate, or high risk of bias based on the overall assessment. Detailed results are provided in Supplementary Tables S2, S3. Risk-of-bias assessment was independently performed by two reviewers (AC and ABT), with disagreements resolved by consensus.

A total of 40 original studies were included in the final analysis, comprising human, animal, and mixed translational evidence. Given the heterogeneity of study designs and outcomes, results were organized into three thematic sections reflecting levels of evidence: mechanistic pathways linking sleep disruption with glial and circadian processes (3.1), experimental evidence connecting sleep disturbance with amyloid–tau pathology (3.2), and human studies examining associations between objective sleep measures, neuroinflammatory markers, and AD-related biomarkers (3.3).

## 3 Results

### 3.1 Mechanistic pathways: glia, circadian regulation, and sleep

The relationship between sleep disruption, glial activation and AD has been extensively explored in both experimental and clinical studies. Chronic sleep disruption leads to profound changes in glial function, particularly microglia and astrocytes, which are key players in neuroinflammation and neurodegeneration.

In animal models, sleep loss induces microglial activation, leading to an inflammatory environment that accelerates proteinopathy, including Aβ and tau. Specifically, sleep fragmentation in P301S and PS19 tau models has been shown to enhance tau phosphorylation and propagation, alongside increased microglial reactivity in the cortex and hippocampus (Martin et al., 2024; Zhu et al., 2018). These findings indicate that sleep disturbances may exacerbate tau pathology, a hallmark of AD, by modulating glial activity and inflammatory responses.

Astrocytes, in addition to microglia, contribute to the brain’s response to sleep loss. Indeed, during slow-wave sleep, they support glymphatic clearance of metabolic waste, including Aβ and tau. Chronic sleep deprivation impairs this clearance mechanism, reducing the exchange of cerebrospinal fluid (CSF) and interstitial fluid and promoting the accumulation of neurotoxic proteins (Bellesi et al., 2017; Lee et al., 2023). Recent evidence further suggests that restoration or receptor modulation of aquaporin-4 may partially reverse sleep restriction-induced glymphatic dysfunction and cognitive impairment, highlighting the therapeutic relevance of sleep-dependent clearance pathways (Sun et al., 2025; Gao et al., 2025). Supporting the central role of astrocytes in sleep-dependent neuroprotection, optogenetic modulation of astrocytic activity restored slow-wave oscillatory dynamics and attenuated AD-related pathological changes in experimental models, further strengthening the link between astrocytic function, sleep physiology, and neurodegeneration (Lee et al., 2023; Zhao et al., 2024a). Sleep disruption, therefore, not only leads to microglial activation but also impairs astrocytic function, which collectively accelerates neurodegeneration.

Circadian regulation further modulates glial reactivity and amyloid dynamics. In particular, experimental inhibition of REV-ERB nuclear receptors, key regulators of circadian transcription, stimulated microglial Aβ clearance and reduced plaque deposition in 5XFAD mice (Lee et al., 2020), supporting a mechanistic link with clock-gene signaling and microglial function. Additional mechanistic evidence indicates that APP-mediated intracellular signaling may rescue sleep impairment and blood–brain barrier leakage in AD mouse models, suggesting a direct interaction between amyloid-related pathways, sleep regulation, and neurovascular integrity (Puech et al., 2026).

**Figure 2:**
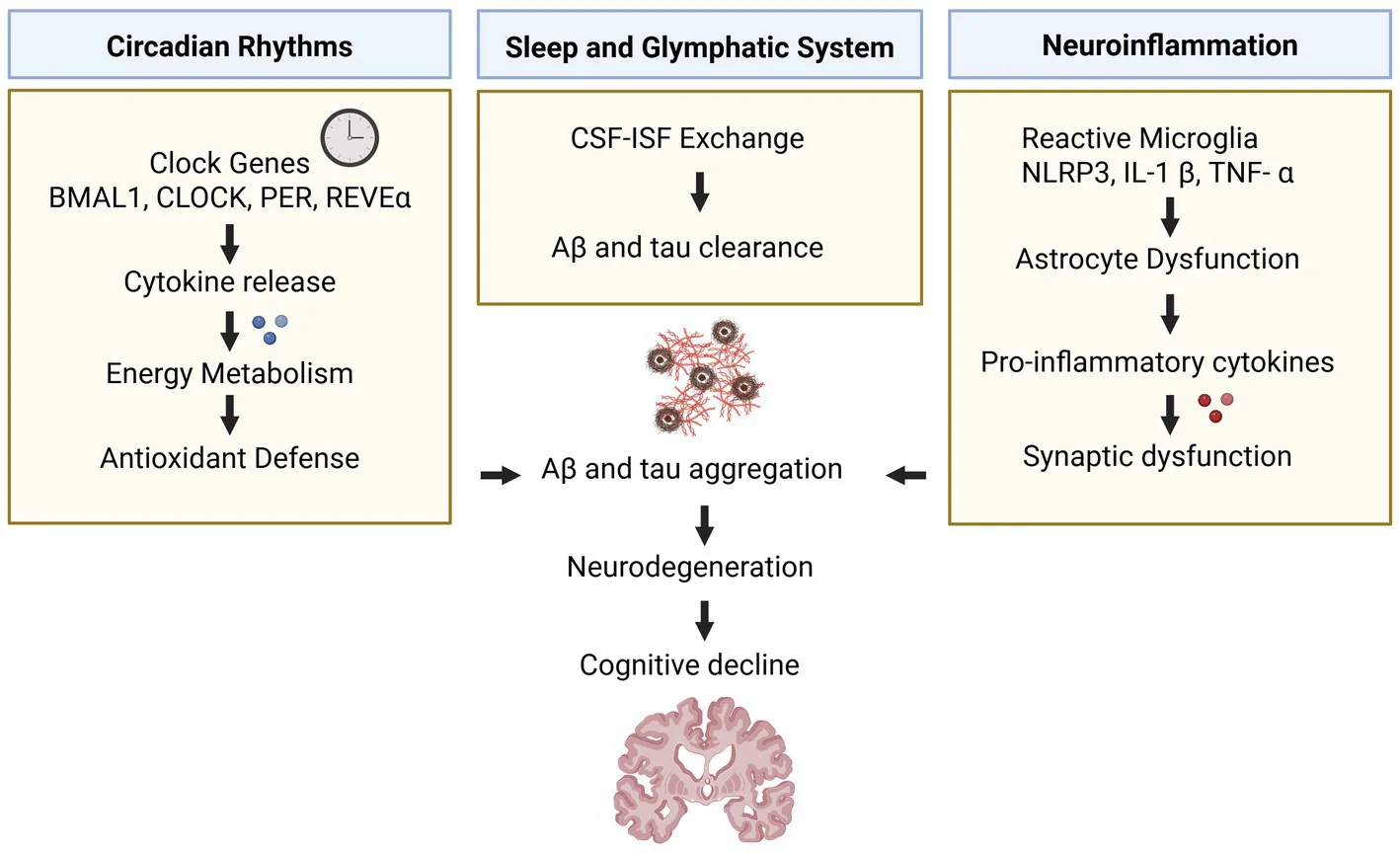
Schematic representation of the pathways linking chronic sleep disruption to the progression of AD. Sleep disturbances, including reduced slow-wave sleep, sleep fragmentation, and circadian misalignment may contribute to inflammatory and metabolic stress, potentially contributing to Aβ and tau accumulation. This cascade leads to glial activation (microglia and astrocytes), neurodegeneration, and cognitive decline. Biomarkers such as CSF or plasma Aβ, tau, and NfL, alongside sleep electroencephalographic measures, have the potential to serve as diagnostic and predictive tools in AD. Created by the authors using BioRender.

Moreover, experimental animal studies have shown that misalignment of circadian rhythms exacerbates inflammatory tone and neuroinflammatory gene expression (Pan et al., 2019; Wisor et al., 2011; Zhan et al., 2019; Zhao et al., 2026) Disruption of the circadian sleep–wake cycle, indeed, has been linked to increased levels of pro-inflammatory cytokines and neuroinflammatory markers, suggesting that maintaining circadian alignment may be critical in preventing or mitigating neurodegenerative processes (Zhao et al., 2026) (Figure 2).

### 3.2 Sleep disruption, amyloid–tau pathology, and immune activation

Across the included experimental studies, sleep disruption—primarily induced through sleep deprivation, sleep restriction, or chronic sleep fragmentation paradigms—was consistently associated with the exacerbation of AD-related pathological processes (Niazi et al., 2025). Evidence from transgenic models indicates that disturbed sleep promotes both Aβ accumulation and tau-related pathology, with several studies demonstrating increased plaque burden, enhanced neuroinflammatory responses, and accelerated neurodegenerative changes following chronic sleep disruption (Liu et al., 2023; Parhizkar et al., 2023; Arjmandi-Rad et al., 2022; Hitrec et al., 2024; Martin et al., 2024; Zhu et al., 2018). Recent findings further suggest the existence of sex-specific vulnerability, with chronic sleep deprivation differentially accelerating AD-related pathological changes in male and female models (Wear et al., 2026).

Sleep fragmentation emerged as a particularly relevant mechanism. It was associated with increased amyloid burden and activation of molecular pathways shared with neurodegeneration (Du et al., 2025; Duncan et al., 2022; Wang et al., 2021; Xie et al., 2020; Hawkins et al., 2025). Importantly, evidence from young wild-type mice suggests that chronic sleep fragmentation may induce neuroinflammatory and cognitive alterations even in the absence of established AD pathology, supporting a potential contributory role of persistent sleep disruption in early neurodegenerative processes (Ba et al., 2021). In parallel, environmental circadian stressors such as dim light exposure during the night have been shown to promote neuroinflammation and AD-like neuropathological changes in APP knock-in models, further highlighting the importance of circadian integrity for brain health (Duncan et al., 2025).

A recurring finding across experimental models was the activation of innate immune pathways. Indeed, sleep deprivation enhanced microglial reactivity, promoted NLRP3 inflammasome activation, increased pro-inflammatory cytokine expression, and facilitated amyloid accumulation (Parhizkar et al., 2023). Chronic sleep restriction was likewise associated with synaptic loss, neuroinflammation, and potentiation of Aβ-induced memory impairment (Kincheski et al., 2017). Astrocytic dysfunction also appeared to contribute to these processes, with sleep disruption promoting oxidative stress, inflammatory signaling, and alterations in glial homeostatic functions (Drew et al., 2023; Niazi et al., 2024; Zhao et al., 2024b). Consistent with these observations, studies in zebrafish models demonstrated that repeated sleep deprivation aggravates AD-related neuropathology through alterations in O-GlcNAc cycling and inflammatory pathways (Park et al., 2023). As additional support for this, a study in APP transgenic mice using optogenetic stimulation of GABAergic interneurons to restore Non-REM slow-wave activity and normalize sleep fragmentation demonstrated enhanced microglial phagocytic activity and reduced Aβ burden (Zhao et al., 2023).

Overall, the available translational evidence supports a close association between experimentally induced sleep disruption, central neuroinflammatory activation, and the worsening of AD-related pathology. Nevertheless, the majority of mechanistic evidence comes from preclinical models, where sleep disruption has been shown to promote microglial activation, Aβ accumulation, and tau-related pathology (e.g., Parhizkar et al., 2023; Duncan et al., 2022; Zhu et al., 2018). However, caution is warranted when extrapolating these findings to human disease, as translational validation remains limited.

### 3.3 Sleep and Neuroinflammatory biomarkers in AD

Among the human studies included in this review, objective sleep measures—primarily polysomnography (PSG), EEG-derived sleep depth metrics, and sleep fragmentation indices—were consistently associated with biomarkers of AD pathology and neuroinflammatory processes. Experimental disruption of slow-wave sleep was shown to acutely increase CSF Aβ concentrations, providing direct evidence that sleep physiology can influence AD-related biomarker dynamics (Ju et al., 2017). Similarly, reduced sleep depth was associated with higher AD biomarker burden in CSF, while alterations in slow-wave activity correlated with markers of synaptic dysfunction, neuroinflammation, and tau-related pathology (Olsson et al., 2018; Targa et al., 2021; Mander et al., 2022).

Beyond amyloid and tau pathology, human studies have highlighted glial activation as a potential intermediary mechanism linking sleep disruption to neurodegeneration. Altered sleep patterns have been associated with changes in CSF AD biomarkers, including reduced Aβ42 levels and increased glial-related inflammatory markers such as YKL-40 and sTREM2 (Olsson et al., 2018). Moreover, sleep fragmentation has been linked to astrocytic activation and cognitive decline in older adults with and without AD (Wu et al., 2023), while alterations in sleep architecture have been associated with inflammatory signatures, tau pathology, and impaired memory performance (Mander et al., 2022). Complementary experimental findings in 5XFAD mice suggested that astroglial activation and sleep architecture disturbances may emerge before overt amyloid deposition and cognitive decline (Zhang et al., 2025). Additional evidence was provided by studies examining astrocyte-derived and CNS-relevant exosomal biomarkers. Sleep-related changes in astrocytic markers appeared to be modulated by APOE ε4 status in cognitively unimpaired individuals, suggesting an interaction between genetic susceptibility and sleep-dependent neuroinflammatory pathways (Zhu et al., 2025). Furthermore, complement proteins and astrocyte-derived exosomal markers were associated with both objective sleep metrics and cognitive performance (Baril et al., 2024; Li et al., 2023). Sleep fragmentation and markers of microglial aging were likewise associated with worse cognitive outcomes (Kaneshwaran et al., 2019; Wu et al., 2023), further supporting a link between disturbed sleep, glial dysfunction, and cognitive decline.

**Figure 3:**
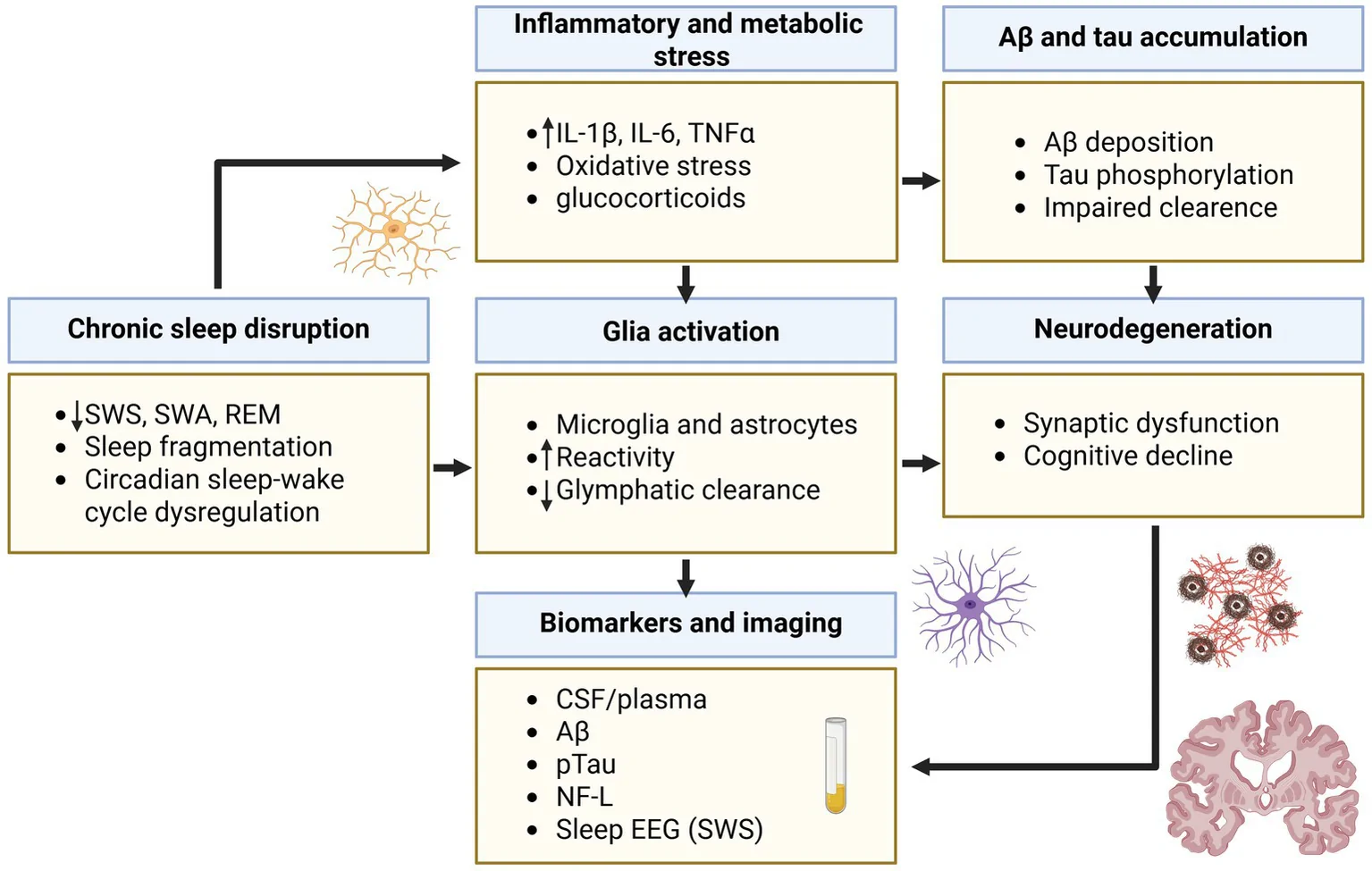
Diagram illustrating the interaction between circadian rhythms, sleep and neurodegeneration in AD. Disruption of circadian regulation has been associated with alterations in cytokine signaling, energy metabolism and antioxidant defenses. This can lead to neuroinflammatory responses, exacerbating glial activation and Aβ/tau aggregation. These disruptions affect cognitive function and accelerate neurodegeneration. Created by the authors using BioRender.

Overall, the available human evidence suggests that objective alterations in sleep architecture are associated with AD-related biomarkers and CNS-relevant neuroinflammatory processes. However, considerable heterogeneity in biomarker selection, sleep assessment methodologies, and study design limits direct comparability across studies. Moreover, the predominance of cross-sectional evidence precludes definitive conclusions regarding the temporal and causal relationships between sleep disruption, neuroinflammation, and AD pathology (Figure 3).

### 3.4 Risk of bias assessment

Risk of bias was assessed using the Newcastle–Ottawa Scale (NOS) for human observational studies and the SYRCLE Risk of Bias tool for animal studies. Translational studies containing experimental animal components were evaluated using the SYRCLE framework according to their predominant methodological design.

Among the nine human studies, NOS scores ranged from 7 to 9, and all nine studies were classified as having a low risk of bias (NOS ≥ 7). Overall, study quality was generally high, with adequate participant selection, objective assessment of sleep measures, and standardized evaluation of biomarker outcomes. The main methodological limitations were the predominance of cross-sectional designs and the potential for residual confounding, which may limit causal inference.

Among the thirty animal studies, the overall risk of bias was judged as moderate. Baseline characteristics, outcome reporting and completeness of data were generally adequately described. However, reporting of methodological safeguards such as sequence generation, allocation concealment, random housing and blinding procedures was frequently incomplete or unclear. Consequently, several SYRCLE domains were rated as unclear risk of bias rather than low risk. These limitations are common in preclinical neuroscience research and should be considered when interpreting mechanistic findings.

One translational study was also judged to be at overall moderate risk of bias. Similar to the animal literature, the principal limitations related to incomplete reporting of randomization procedures, allocation concealment and blinding methods. Nevertheless, outcome assessment and reporting were generally adequate across studies.

Detailed risk-of-bias assessments are provided in Supplementary Tables S2, S3.

## 4 Discussion

### 4.1 Summary of findings

We systematically review the intricate relationship between sleep architecture pathological changes, neuroinflammation, and AD. The included studies underscore the bidirectional nature of these interactions, with sleep disruption potentially contributing to the progression of Aβ and tau pathology and glial activation in both animal models and human cohorts. These observations highlight sleep physiology as a potential biological target for future investigation, although disease-modifying effects remain unproven in humans. The available evidence highlights the central role of microglial activation and astrocytic function in mediating the interaction between sleep disruption and AD-related pathology. The reviewed literature also underscores the importance of sleep quality in supporting glymphatic clearance mechanisms involved in the removal of neurotoxic proteins. Collectively, these observations suggest that the interaction between sleep physiology and central inflammatory pathways may represent a promising target for early intervention in AD.

### 4.2 Therapeutic implications

Given the growing evidence linking sleep disruptions with neurodegeneration and neuroinflammation, future therapies may benefit from targeting sleep quality as part of a broader neuroprotective strategy. Pharmacological approaches, such as orexin-receptor antagonists and melatonin supplementation, have shown promise in improving sleep architecture and reducing neuroinflammatory signals in preclinical models. Maintaining the regularity of the sleep–wake cycle and preserving sleep quality and continuity may mitigate systemic inflammation, thereby reducing the possibility of triggering the AD pathological cascade of events affecting brain health (Irwin and Opp, 2016; Riemann et al., 2017).

Moreover, strategies to improve glymphatic clearance, such as sleep manipulation and exercise, could help reduce amyloid burden in the brain (Mander et al., 2022). Although sleep metrics may eventually contribute to risk stratification and biomarker profiling, current evidence is insufficient to support their routine use in clinical decision-making.

### 4.3 Limitations

While this review provides a comprehensive synthesis of the current literature, several limitations should be acknowledged. First, substantial heterogeneity was observed across the included studies with respect to study populations, disease stages, animal models, sleep assessment methodologies, neuroinflammatory biomarkers, AD-related outcomes, and analytical approaches, limiting direct comparability across studies. This methodological and biological heterogeneity also precluded the performance of a meaningful meta-analysis, as pooling highly diverse datasets could have generated potentially misleading summary estimates.

The majority of included preclinical studies relied on transgenic or knock-in rodent models that reproduce selected aspects of AD pathology over a relatively compressed timeframe, frequently in combination with experimentally imposed sleep disruption. In contrast, sporadic human AD develops over decades within an ageing brain and is characterized by chronic and heterogeneous neuroimmune activation. These temporal and biological differences limit direct extrapolation from experimental models to human disease (Mary et al., 2024). At the cellular level, single-cell studies have identified disease-associated microglia (DAM) and activated-response microglia (ARM) in mouse AD models; however, their transcriptional programs only partially overlap with the more heterogeneous microglial states observed in human AD, including signatures described as human Alzheimer’s microglia (HAM). Likewise, reactive astrocytes display context-, stage-, and species-dependent transcriptional and functional states that are not fully reproduced by experimental models. Therefore, glial responses observed in rodents should not be considered direct equivalents of those occurring in human AD, and mechanistic extrapolation should remain cautious (Chen and Colonna, 2021; Mary et al., 2024; Patani et al., 2023).

The available human evidence remains limited by the predominance of cross-sectional designs, relatively small sample sizes, and variable control of important confounders. Consequently, definitive conclusions regarding causal relationships between sleep disruption, neuroinflammation, and AD pathology cannot yet be drawn. Because many available studies are cross-sectional, the directionality of these associations remains uncertain, making it difficult to determine whether sleep disturbances precede neurodegenerative processes or represent an early manifestation of AD pathology. This uncertainty has been emphasized by the 2024 Lancet Commission on dementia prevention, which concluded that current evidence is insufficient to consider sleep disturbance a confirmed modifiable risk factor for dementia, partly due to the possibility of reverse causation and the heterogeneity of sleep assessments across studies (Livingston et al., 2024). Consistently, a recent systematic review examining the relationship between sleep duration and fluid AD biomarkers reported heterogeneous and often inconsistent associations across studies, highlighting substantial variability in sleep definitions and biomarker panels (Young et al., 2026).

Additional limitations include the restriction to English-language publications, which may have excluded relevant studies published in other languages and may therefore represent a source of selection bias. Furthermore, restricting the literature search to title/abstract fields may have resulted in the omission of potentially relevant studies indexed using different terminology or reported only within the full text.

### 4.4 Future directions

Together, these findings support a conceptual framework in which sleep disruption, glial activation, and AD pathology interact within a dynamic and potentially self-reinforcing biological network. Future research should aim to integrate objective sleep measurements, neuroinflammatory markers, and AD biomarkers in longitudinal studies of human populations. Such studies would help to clarify the temporal relationship between these factors and their impact on the appearance or progression of the disease. Furthermore, the development of standardized methodologies for sleep assessment, neuroinflammation measurement, and AD biomarker detection is essential to improve the comparability of studies.

Emerging technologies, such as AI-based multimodal data integration and digital twin models, could play a critical role in personalized medicine for AD. By incorporating sleep, inflammatory markers, and neuroimaging data, these tools could help predict disease trajectories and tailor interventions to individual patients.

## Data Availability

The original contributions presented in the study are included in the article/Supplementary material, further inquiries can be directed to the corresponding author.
